# Manipulation of Innate Immunity for Cancer Therapy in Dogs

**DOI:** 10.3390/vetsci2040423

**Published:** 2015-11-30

**Authors:** Daniel Regan, Steven Dow

**Affiliations:** 1 Flint Animal Cancer Center, Department of Clinical Sciences, Colorado State University, Ft. Collins, CO 80525, USA; E-Mail: Daniel.Regan@colostate.edu; 2 The Center for Immune and Regenerative Medicine, Department of Clinical Sciences, Colorado State University, Ft. Collins, CO 80525, USA

**Keywords:** innate immunity, cancer, treatment, macrophage, monocyte, dog

## Abstract

Over the last one to two decades, the field of cancer immunotherapy has rapidly progressed from early preclinical studies to a successful clinical reality and fourth major pillar of human cancer therapy. While current excitement in the field of immunotherapy is being driven by several major breakthroughs including immune checkpoint inhibitors and adoptive cell therapies, these advances stem from a foundation of pivotal studies demonstrating the immune systems role in tumor control and eradication. The following will be a succinct review on veterinary cancer immunotherapy as it pertains to manipulation of the innate immune system to control tumor growth and metastasis. In addition, we will provide an update on recent progress in our understanding of the innate immune system in veterinary tumor immunology, and how these gains may lead to novel therapies for the treatment of cancer in companion animals.

## 1. Introduction

Immunotherapy for cancer has rapidly moved from a research concept to successful clinical reality in cancer treatment for humans in the span of just 10 years [1,2,3,4,5,6]. Several major breakthroughs are driving the current excitement around cancer immunotherapy, including the discovery that blockade of immune checkpoint molecules can stimulate durable tumor remissions, as well as the administration of engineered T cells designed to target specific antigens on tumor cells. In addition, our understanding of the critical role of the innate immune system in regulating adaptive immune responses to cancer has also increased rapidly [7,8,9]. Thus, we are poised now to add immunotherapy as a fourth major pillar of cancer therapy, in addition to surgery, chemotherapy, and radiation therapy. The following review will provide historical perspective on the use of biological response modifiers to activate innate immunity for tumor control, as well as discuss more recent studies using molecules targeting specific pathways in innate immune activation to induce non-specific anti-tumor immunity.

## 2. Dual Roles of Innate Immunity in Cancer Control

**Figure 1:**
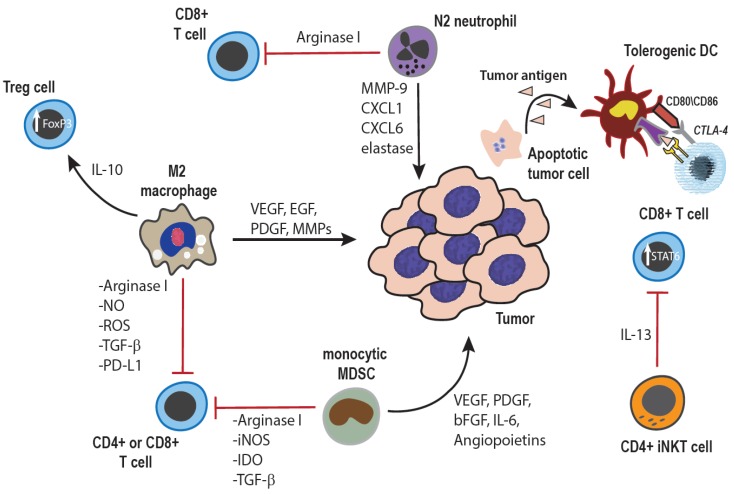
**Tumor-promoting effects of innate immune cells:** Cells of the innate immune system can promote tumor growth through both direct interactions with tumor cells, as well as indirectly through modulation of the adaptive immune response. Macrophages within the tumor microenvironment can be polarized towards an anti-inflammatory, M2 phenotype, and secrete cytokines, growth factors, and enzymes which promote angiogenesis as well as tumor cell survival, proliferation, invasion, and metastasis [3,25]. Additionally, both macrophages and monocytes can inhibit anti-tumor T cell responses through production of anti-inflammatory cytokines, induction of Treg cells, expression of negative co-stimulatory ligands such as PD-L1 or PD-L2, and depletion of extra-cellular arginine, an amino acid essential for normal T cell function and proliferation [7,14,25,26,27]. Similarly, tumor-associated neutrophils can also be polarized towards a tumor-promoting N2 phenotype, elaborating similar growth factors and pro-angiogenic cytokines, as well as suppressing T cell responses by mechanisms similar to those used by monocytes and macrophages [25,28]. The immune suppressive cytokine milieu within the tumor microenvironment can also inhibit DC maturation, resulting in tumor antigen-specific anergy of T cells [29]. Lastly, specific CD4+ subsets of invariant NK T cells are also known to produce immune suppressive cytokines, which inhibit CD8+ anti-tumor T cell responses [30].

The innate immune system plays a much more nuanced role in cancer immunity than the adaptive immune system (T cells and B cells), which is considered in most cases to actively suppress tumor growth. It is now apparent that the innate immune system can in many cases suppress adaptive immune responses and remove immune checks on tumor growth (Figure 1) [1,2]. In addition, cells of the innate immune system, especially tumor-associated macrophages, can directly interact with tumor cells to stimulate tumor cell growth, genetic instability, and metastasis [3,4,5,6]. Macrophages can also modify the tumor microenvironment to enhance tumor growth, including increasing angiogenesis, stimulating local release of reactive oxygen species, and promoting tumor invasion through extracellular basement membranes. Inflammatory monocytes recruited to sites of early tumor metastases promote the early survival of metastatic tumor cells, in part through enhanced angiogenesis [7,8]. We have reported that increased numbers of circulating monocytes are associated with shorter survival times in dogs with osteosarcoma and animals with B cell lymphoma [9,10]. Thus, on balance macrophages and monocytes can be considered as major drivers of tumor growth and progression.

**Figure 2:**
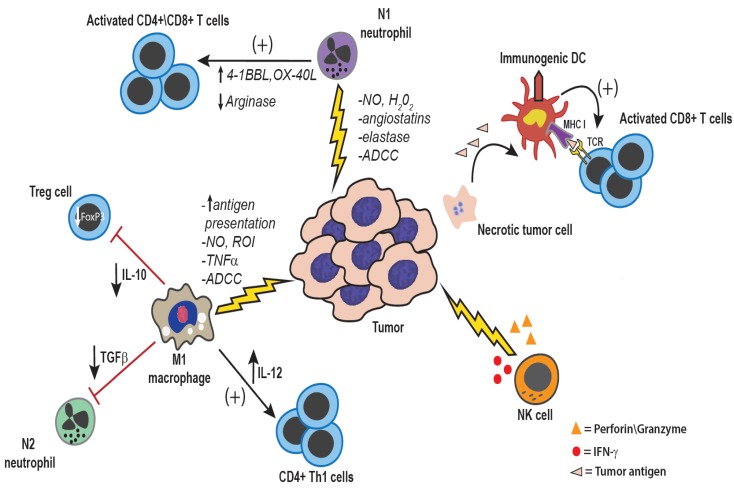
**Anti-tumor effects of innate immune cells:** Both tumor-associated macrophages and neutrophils can be polarized to a more pro-inflammatory anti-tumor phenotype, either inherently within certain tumor types, or through therapeutic manipulation. Direct anti-tumor mechanisms of macrophages and neutrophils are mediated by production of reactive nitrogen and oxygen intermediates, cytokines such as TNF-α, and enzymes such as elastase [25,31]. Additionally, through the production of IL-12, macrophages can activate NK cells as well as induce a Th1 type anti-tumor immune response [32]. NK cells are also potent anti-tumor innate immune effector cells. NK cells are activated in response to reduced expression of MHC I and by ligation of activating receptors such as NKG2D [33]. NK cells mediate direct tumor cell killing via perforin and granzyme, or expression of FasL and TNF-related apoptosis-inducing ligand (TRAIL) [33]. Additionally, NK cells are an important source of IFN-γ within the tumor microenvironment, which serves to activate macrophages, and DCs, and up-regulated MHC I and MHC II expression on tumor cells and antigen-presenting cells, respectively [33].

**Figure 3:**
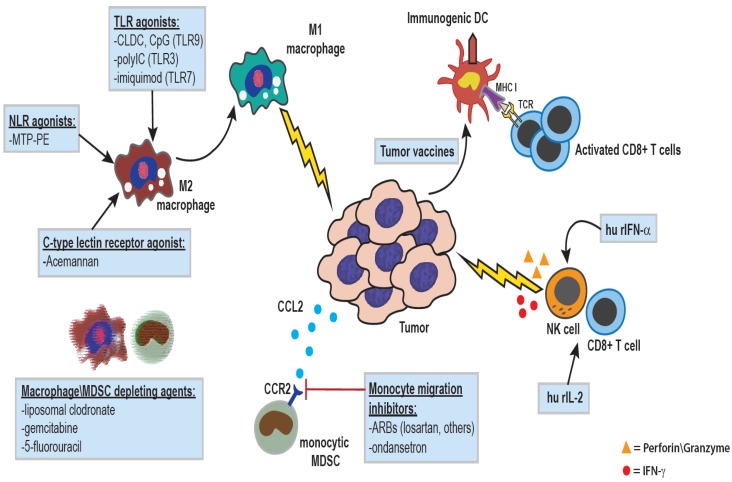
**Therapeutic manipulations of the innate immune system for treatment of cancer:** The administration of agonists for various pattern-recognition receptors, including Toll-like receptors (cationic liposome-DNA complexes (CLDC), pIC, or imiquimod), Nod-like receptors (liposomal muramyl tripeptide), or lectin receptors (acemannan), can result in macrophage activation and polarization towards a pro-inflammatory anti-tumor phenotype. IL-2 is a potent activator of NK and T cells, and human recombinant IL-2 has been used in the treatment of multiple canine cancer types including melanoma, metastatic osteosarcoma, lymphoma, and soft tissue sarcoma. Type I interferons such as IFN-α serve to activate and enhance DC maturation, and increase cytotoxicity of CD8+ T cells and NK cells, and recombinant human IFN-α has been administered to dogs with various epithelial neoplasms. Macrophages and monocytes can also be targeted with various drugs as a means of augmenting tumor angiogenesis and restoring anti-tumor immunity. Drugs such as liposomal clodronate or conventional chemotherapeutics like gemcitabine and 5-fluorouracil can result in systemic depletion of macrophages\monocytes [34], while work in our lab has shown that small molecules drugs such as ondansetron, and angiotensin-receptor blockers like losartan, can function to inhibit monocyte migration.

However, it is also clear that macrophages can be activated therapeutically to control tumor growth (Figure 2 and Figure 3). Earlier studies demonstrated that activated macrophages could kill tumor cells, primarily via the secretion of TNF-α. For example, canine alveolar macrophages activated by the Nod-like receptor (NLR) agonist muramyl tripeptide (MTP) were shown to kill canine osteosarcoma cells *in vitro* [11,12].Other immune activating molecules, including certain Toll-like receptor ligands (TLR), can also induce macrophage killing of tumor cells [13]. Administration of cytokines such as IL-12 and INF-γ can also activate macrophages to become tumoricidal. 

A mixed population of immature myeloid cells (comprised primarily monocytes and neutrophils) collectively known as myeloid derived suppressor cells (MDSCs) contribute in a major way to global suppression of tumor immunity [14,15,16,17,18]. Large numbers of MDSCs are found in cancer patients and individuals with chronic infections [19,20,21]. Expanded circulating populations of MDSCs have been described in dogs with cancer [22,23]. MDSCs infiltrate the bone marrow and blood stream, as well as secondary lymphoid tissues (spleen and peripheral lymph nodes), and tumor tissues, where they potently suppress T cell and NK cell responses [14,15]. The mechanisms by which MDSCs suppress T cells vary by species, but include production of immune suppressive metabolites (e.g., reactive nitrogen and oxygen intermediates), production of immunologically active enzymes (arginase, indoleamine dioxygenase, aminopeptidases), nitrosylation of T cell receptors, production of immune suppressive cytokines (e.g., TGF-β, IL-10) and by production of immune suppressive prostaglandin E [24]. In dogs, MDSCs are reported to suppress T cell function by production of arginase, which leads to local depletion of arginine, an essential amino acid required for normal T cell function [22,23]. Myeloid derived suppressor cells are therefore very attractive targets for immunotherapeutic manipulation of both the innate and adaptive immune systems.

Conventional NK cells, when appropriately activated, can exert powerful tumor suppressive activity (Figure 2) [33,35,36]. For example, *in vivo* administration of molecules that elicit production of type I interferons (e.g., IFN-α and IFN-β) can activate and expand NK cell populations, which control tumor growth by producing IFN-γ and by directly inducing tumor lysis [36]. A subpopulation of NK cells known as Natural Killer T cells (NKT cells) can also be directly activated by administration of the CD1 ligand alpha-galactosyl ceramide. Depletion of NK cells or NK cell dysfunction is associated with increased spontaneous generation of tumors [37,38]. However, not all NK cells control tumor growth, as certain subpopulations of NK cells can also suppress tumor immunity by producing immune suppressive cytokines (e.g., IL-10, IL-13) and promoting the growth of regulatory T cells (Tregs) (Figure 1) [39,40].

The role of neutrophils in the regulation of tumor immunity remains incompletely defined. Recent studies in mice have suggested that neutrophils, similar to tumor-associated macrophages, can be polarized towards either a pro-tumor or anti-tumor phenotype, termed N1 or N2, respectively [28]. Consistent with this paradigm, some studies have demonstrated a protective effect of tumor-associated neutrophils in tumor immunity via stimulation of T cell activation and proliferation, while others have shown that neutrophils may subvert tumor immune responses through mechanisms similar to those reported for MDSCs [25,41,42,43]. Recent studies in dogs suggest that neutrophils may be a major component of the expanded MDSC population observed in dogs with cancer [23]. Our studies and others have found an association between increased numbers of circulating neutrophils and poor outcomes in dogs with lymphoma and OS [10].

## 3. Activation of Innate Immunity for Cancer Immunotherapy Using Biological Response Modifiers

Cancer immunotherapy has a relatively long history in veterinary medicine. In humans, non-specific immunotherapy dates back to the pioneering studies of William Coley in the late 1800s, who demonstrated that injection of live bacteria or bacterial extracts could induce tumor regression in human sarcoma patients [44]. Many of the earliest immunotherapy studies using so-called biological response modifiers were done in dogs with cancer. For example, immunotherapy using live, attenuated *Mycobacterium bovis* (strain Bacillus Calmette-Guerin; or BCG) was conducted in dogs beginning in the 1970s [45,46]. Injection of BCG in rodent models was shown to activate a number of innate immune pathways, including macrophages, monocytes, neutrophils, and NK cells. In dogs, BCG was administered by a variety of routes to dogs with osteosarcoma (OS) to elicit non-specific tumor immunity, with or without administration of irradiated tumor cells as vaccines. Significant increases in survival times in dogs with OS were observed following BCG treatment, and the effects were attributed to activation of macrophage tumoricidal activity [45]. Specifically, the median survival time (MST) of BCG-treated dogs with no radiographic evidence of pulmonary metastasis at time of amputation was greater than 41 weeks (287 days), which was significantly longer than control dogs receiving amputation alone (MST of 11 weeks/77 days) [45], and is comparable to those reported for the current standard of care adjuvant chemotherapies of carboplatin and doxorubicin [47,48,49]. Importantly, 2 of the 6 BCG-treated dogs were still alive with no radiographic evidence of metastasis 322 and 371 days after amputation [45].

## 4. Activation of Innate Immunity by Nod-Like Receptor Antagonists

In the 1980s, a more fully defined cancer immunotherapeutic derived from biological sources was developed. This new compound (MTP-PE) consisted of the Nod-like receptor ligand muramyl tripeptide, delivered within phosphatidyl ethanolamine liposomes [50,51]. MTP was first identified as a bacterial peptide that stimulated neutrophil migration and activation, and later its ability to activate monocytes and macrophages was also discovered. Only recently was the actual receptor for MTP identified. Nod-like receptors (NLRs) are intracellular receptors that recognize diverse ligands, including products of bacterial degradation (e.g., peptidoglycans), viral nucleic acids, uric acid, and even changes in intracellular K^+^ concentration [52,53].

*In vitro* incubation of tumor cells and PBMC with L-MTP-PE induced tumor cytolysis, in a process that was shown to be dependent on TNF-α production by monocytes and macrophages. Intravenous administration of L-MTP-PE to dogs induced rapid release of TNF-α into circulation, and also induced TNF-α production by alveolar macrophages [54]. Clinical trials of L-MTP-PE were initiated in dogs with OS, initially as a single agent for adjuvant therapy to prevent metastasis, and was shown to significantly prolong survival compared to amputation alone, with a MST of 222 days for dogs receiving L-MTP-PE, as compared to 77 days for dogs receiving control, empty liposomes [11,12,55]. When administered in succession with cisplatin chemotherapy, L-MTP-PE further improved median survival times to 14.4 months (~439 days), as compared to 9.8 months (~299 days) for dogs receiving cisplatin alone; however, when the two drugs were administered concurrently, the additive benefit was lost [12]. L-MTP-PE is now approved for treatment of pediatric OS in Europe, but has not been approved in the US and is no longer available for routine clinical use.

## 5. Activation of Anti-Tumor Immunity by Biological Molecules

The first Toll-like receptors (TLRs) were discovered in the 1990s and an explosion of research led to the identification of at least 11 different TLRs. TLRs function primarily to recognize pathogen molecules, including viral, bacterial, and fungal pathogens, and are expressed by many different cell types, especially antigen presenting cells (macrophages, monocytes, dendritic cells, neutrophils) of the innate immune system. Early immunotherapy studies by William Coley in the late 1800s using complex mixtures of bacterial products to stimulate innate immune activation provided the first evidence that bacterial products could trigger immune activation sufficient to cause tumor regression or sustained tumor stasis [44]. We now know that the activity of Coley’s toxin was mediated by simultaneous activation of multiple TLRs, which provides a very potent signal to innate immune cells [56].

## 6. Immunotherapy by Activation of C-Type Lectin Receptors Using Plant Extracts

One of the earliest cancer immune therapeutics evaluated in veterinary medicine consisted of extracts of the Aloe plant, known as Acemannan. This product consists of mannan polymers interspersed with O-acetyl groups. Though not proven, it is likely this compound activates innate immune cells via the dectin receptor, a C-type lectin known to bind to beta-linked glucans from plants and fungi [56]. Clinically, Acemannan was shown to induce tumor regression following direct intra-tumoral injection in dogs with fibrosarcoma, and to induce systemic immune activation following i.p. administration [57,58]. However, there are no data to suggest that the drug has a systemic effect in preventing local tumor recurrence or tumor metastasis. Additionally, it should be noted that while acemannan showed no direct toxicity following repeated injections in normal tissues, the marked intra-tumoral inflammation and secondary necrosis elicited by the drug can be extensive, subsequently requiring surgical excision of the tumor [58]. Thus, location of the primary tumor and complexity of surgical removal should be considered prior to initiating therapy with acemannan. The drug is still commercially available from Carrington Laboratories (now marketed by VPL, Inc., Phoenix, AZ, USA).

## 7. Activation of Anti-Tumor Immunity in Dogs with TLR Agonists

TLR-9 is a key sensor of bacterial infections (both Gram^+^ and Gram^−^) and recognizes certain CpG motifs present in all DNA of bacterial origin [56]. The TLR9 molecule is expressed in an endosomal location, such that only internalized bacterial DNA is sensed. TLR9 can be activated by administration of short oligonucleotides containing CpG motifs (CpG oligos) and activation leads to strong activation of DCs, monocytes, and macrophages, with release of a variety of pro-inflammatory cytokines, including TNF-α, IL-12, IL-6, and type I interferons. Immunotherapy with CpG oligos demonstrated impressive anti-cancer activity in mouse models in the late 1990s [59,60,61,62]. However, human clinical trials failed to demonstrate sufficient activity and commercial products for cancer immunotherapy have not been vigorously pursued. However, more recent studies suggest that local intra-tumoral injection of CpG oligonucleotides, combined with local administration of antibodies that inhibit immune checkpoint molecules, may have a new role to play in innate immune control of cancer [63].

Larger DNA molecules (e.g., plasmid DNA) can also activate TLR9 when complexed first to charged liposomes, which facilitate intracellular entry and endosomal uptake. These cationic liposome-DNA complexes (CLDC) have been shown to potently stimulate innate immunity and NK cell mediated anti-tumor activity in mouse models [64,65]. Importantly, CLDC have also demonstrated an ability to strongly activate innate immunity in dogs following i.v. administration. For example, i.v. infusion of labeled CLDC resulted in significant uptake and activation of circulating monocytes, characterized by strong up regulation of MHC class II and CD86 expression along with activation of NK cells, as evidenced by enhanced *in vitro* spontaneous cytolysis of MHC-mismatched target cells [66]. Other effects of CLDC infusion in dogs include binding to tumor blood vessels, inhibition of tumor angiogenesis and up regulation of MHCII expression by T cells [67]. Clinically, CLDC infusion was shown to significantly prolong survival in dogs with established OS pulmonary metastases, with CLDC-treated dogs having a MST of 82 days, as compared to median survival times of 58 days for untreated control dogs, and 61 days for dogs receiving cisplatin and/or doxorubicin in the metastatic setting [66,68]. Additionally, CLDC infusion was also shown to induce tumor growth stabilization in dogs with soft tissue sarcoma, with 8 of 13 dogs exhibiting stable disease, and one dog each exhibiting complete and partial responses [67]. Other studies have shown that s.c. administration of CLDC can trigger full regression of adult-onset papillomatosis in dogs [69]. Commercially, CLDC is now approved in Europe as an immunotherapeutic for use in poultry and is currently being investigated as a mucosal immune stimulant in companion animals.

The use of TLR 3 agonists has also been explored in cancer immunotherapy in rodent models and in limited human clinical trials. These studies have primarily utilized a synthetic analog of single-stranded RNA known as polyinosinic-polycytidylic acid, or pIC. This molecule activates the intracellular TLR3 receptor and triggers release of type I interferons (IFN-α and IFN-β) as well as other pro-inflammatory cytokines. Complexes of pIC and cationic liposomes are also capable of activating innate immunity in cats and dogs [70].

**Table 1:** Anti-Tumor Immune Effector Molecules.

Cytokine\Immune Molecule	Cellular Source	Anti-Tumor Mechanism(s)
*IFN-γ*	- Predominately CD4+ and CD8+ T cells and NK cells [71]	- Direct anti-proliferative, anti-angiogenic, and pro-apoptotic effects on tumor cells [71,72] - Increases MHC I expression on tumor cells and APCs for enhanced tumor immune recognition and killing [71,72] - Enhanced tumoricidal and phagocytic activity of macrophages [71,72] - Promotes differentiation and activation of a Th1 immune response [71,72]
*IL-12*	- Professional APCs (macrophages and dendritic cells) [73]	- Activation, enhanced cytotoxicity, and induction of IFNγ production by NK cells and CD4+ and CD8+ T cells [73] - Promotes differentiation and activation of a Th1 immune response [73] - Enhanced ADCC [73]
*IL-2*	- Activated CD4+ and CD8+ T cells, NK and NK T cells [74]	- Stimulates survival, proliferation, and activation of CD4+ and CD8+ T cells and NK cells [74] - Enhanced cytotoxicity and cytokine production by CD4+ and CD8+ T cells and NK cells [74]
*IFN-α/β*	- Dendritic cells - Macrophages - Non-immune cells (epithelial cells, fibroblasts) [75]	- Stimulates DC maturation and migration to LNs; Enhances their ability to process and present dead tumor cell antigens [75] - Increased cytotoxicity of NK and CD8+ T cells [75] - Increased survival of memory CD8+ T cells [75] - Increased pro-inflammatory cytokine release by macrophages [75] - Decreased suppressive function of Treg cells [75]
*TNF-α*	- Primarily activated macrophages, T cells, and NK cells [76,77] - Also stromal cells such as fibroblasts [76,77]	- Direct induction of apoptosis in tumor cells [76] - Important effector molecule for CD8+ T cell and NK cell mediated tumor cell cytotoxicity [77] - Induces apoptosis of endothelial cells and disrupts the tumor vasculature [77,78] * TNF-α can also have tumor-promoting effects through activation of NFκB, which enhances transcription of genes associated with tumor cell survival, proliferation, invasion, and metastasis [76]
*Perforin and granzyme*	- CD8+ T cells and NK cells [79]	- Mediate direct tumor cell killing by activation of intrinsic apoptosis via proteolysis of anti-apoptotic proteins or direct cleavage of caspase 3 [79] - Increase ROS production resulting in oxidative damage [79] - Can active caspase 1 to promote release of IL-1β [79] - Extracellular granzymes can activate macrophages to produce pro-inflammatory cytokines [79]
*FasFasL*	- Predominately CD8+ T cells, NK cells, and tumor cells [80]	- FasL expression by CD8+ T cells mediates extrinsic apoptosis of Fas expressing tumor cells via activation of caspase 8 [80] - Tumor cell FasL expression is a means of immune escape via induction of apoptosis of Fas expressing CD4+ and CD8+ T cells [80]
*TRAIL*	- NK cells, CD4+\CD8+ T cells [81] - Monocytes, macrophages, DCs	- Engages TRAIL receptors 1 and 2 on tumor cells to induce apoptosis via both extrinsic and intrinsic pathways

## 8. Innate Immune Activation by Recombinant Cytokines

The innate immune system can also be activated by administration of cytokines, including IL-2, IL-12, IFN-γ, IFN-α, and TNF-α (Table 1). TNF-α was one of the first cytokines identified with antitumor activity, and was widely investigated as a stand-alone immunotherapeutic. However, systemic administration of TNF-α was accompanied by substantial toxicity, which precluded its use in humans or in companion animals. IL-2 was investigated in dogs as a means of activating spontaneous NK cell activity [82,83,84]. These studies utilized human recombinant IL-2 and demonstrated *in vitro* activation of NK cell activity. A safe dose for i.v. administration of huIL-2 was also determined, though formal clinical trials in cancer-bearing animals were not pursued. Inhalational administration of human IL-2 has also been shown to generate significant antitumor activity in dogs with lung metastases [85,86]. Interferon-α has been widely used for immunotherapy of cancer in dogs and in cats. Studies in rodents and humans indicate that IFN-α is capable of triggering NK cell proliferation and activation, which likely accounts for its antitumor activity. While IFN-α is FDA approved for the treatment of various human cancers, including chronic myeloid leukemia, melanoma, and multiple myeloma [75], randomized clinical trials have not yet been conducted in dogs or cats treated with IFN-α, though the drug is anecdotally administered for certain types of canine and feline cancers, including squamous cell carcinoma and papillomatosis. Currently, a canine IFN-γ product is not available, and human IFN-γ does not cross react with dog or cat innate immune cells. 

## 9. Reversal of Immune Suppression by Macrophage Depletion or Monocyte Migration Blockade

Most previous work in tumor immunotherapy has focused on direct activation of innate immune cells to elicit tumor immune control. However, it is now clear that certain myeloid cells (especially tumor associated macrophages and circulating immature monocytes and neutrophils) can exert a profound suppressive influence on tumor immunity [14,15,17]. In cancer patients, low level sustained inflammation drives the release of immature monocytes and neutrophils from the bone marrow, and also prevents their maturation once they reach peripheral tissues [24]. The primary immunological targets of these so-called myeloid derived suppressor cells (MDSCs) are T cells and NK cells, though MDSCs can also directly affect tumor behavior as well. For example, MDSCs interfere with multiple steps in the T cell activation and expansion cascade, through a variety of mediators including production of reactive nitrogen and oxygen intermediates, production of indoleamine dioxygenase, arginase, and immune suppressive cytokines (TGF-β, IL-10, VEGF) [24] (Figure 1).

These expanded populations of MDSCs therefore make an attractive target for immunotherapeutic intervention in cancer; to help restore T cell and NK cell anti-tumor function. For example; studies in rodent models have demonstrated that the biochemical pathways mediating immune suppression by macrophages can be interrupted by specific pathway inhibitors; though these inhibitors have not been evaluated clinically in dogs with cancer [87]. Alternatively; MDSCs can also be targeted for specific elimination pharmacologically. Currently; the most effective methods of eliminating MDSCs (and tumor-associated macrophages and monocytes) take advantage of the fact that these cells are highly phagocytic; and actively phagocytose particles in the size range of 100 nm to 1 μm in diameter. Thus; when the bisphosphonate drug clodronate; which induces macrophage apoptosis by competing for ATP energy stores; is encapsulated within liposomes and administered to animals; macrophages phagocytose the liposome-encapsulated drug; resulting in cytoplasmic drug release and rapid induction of macrophage apoptosis [88]. Our studies in rodent models demonstrated that treatment of tumor-bearing mice with liposomal clodronate induced efficient depletion of MDSCs and tumor macrophages [89]. Importantly; MDSC depletion with liposomal clodronate also induced tumor growth arrest; associated with spontaneous T cell and NK cell activation.

Liposomal clodronate (LC) has also been evaluated as a cancer immunotherapeutic in dogs. In one study, i.v. infusion of liposomal clodronate was shown to induce tumor regression in some dogs with histiocytic sarcoma that had previously failed conventional prednisone and/or lomustine chemotherapy, with 1 of the 5 treated dogs surviving for five months following LC treatment [90]. *In vitro*, liposomal clodronate was also shown to induce direct killing of canine histiocytic sarcoma cells, by virtue of their phagocytic properties, whereas most tumor cells of other lineages (which are not phagocytic) were not affected. In a second study, liposomal clodronate was also administered to dogs with soft tissue sarcomas and effects on tumor macrophages and tumor angiogenesis were assessed prior to treatment and again following 3 treatments [91]. We found that treatment with liposomal clodronate was associated with significant depletion of tumor-associated macrophages, as well as a significant reduction in tumor microvessel density; however, these reductions in tumor macrophages and angiogenesis did not translate to objective tumor responses [91]. Of not, adverse effects related to liposomal clodronate treatment in these two studies were limited and included fever, and transient neutrophilia [90,91]. Thus, these studies illustrate that macrophages and other immunosuppressive myeloid cell populations can be efficiently depleted in dogs by liposomal clodronate.

An alternative strategy for eliminating tumor macrophages is to selectively block the migration and recruitment of inflammatory monocytes to tumor tissues. Tumor production of certain chemokines, especially the chemokine CCL2, is a major driver of monocyte mobilization of inflammatory (CCR2^+^) monocytes from the bone marrow and recruitment into tissues, where the monocytes mature into tumor macrophages, which support the growth of tumor metastases [7]. Sustained administration of drugs that block the CCR2 receptor can over time induce macrophage depletion and slow tumor growth in rodent models (Dow, S., unpublished data). Moreover, we have recently observed that highly metastatic tumors in dogs (e.g., hemangiosarcoma and OS) are associated with intense monocyte infiltrates and local production of CCL2 (Regan, D., *et al.*, manuscript in preparation). Importantly, we have also observed that certain classes of drugs (e.g., angiotensin-receptor blocking agents such as losartan) can also block CCL2 dependent migration of canine monocytes *in vivo* and *in vitro*. Thus, in the future it may be possible to combine myeloid targeted immunotherapeutics with other treatment modalities (e.g., chemotherapy or radiation therapy) to help augment anti-tumor immunity [92].

## 10. Conclusions and Implications

Recent successes in immunotherapy for treatment of cancer in humans have generated a considerable reawakening of interest in tumor immunotherapy in veterinary medicine as well. At present, the best opportunities for use of immunotherapy in treatment of companion animal cancer include administration of activators of innate immunity, including both TLR and NLR agonists. In addition, alternative routes of delivering activating ligands are likely to figure more prominently in cancer immunotherapy. There is also a great deal of interest in the use of monoclonal antibodies targeting immune checkpoint molecules such as PD-1 and CTLA-4, which will hopefully become available in the near future. The combination of innate immune targeted therapy with cancer vaccines and checkpoint molecule inhibitors will undoubtedly be explored as well. Therefore it is not unrealistic to expect that immunotherapy will soon gain accepted status as the fourth major component of tumor therapy in companion animals as wells as in humans.
